# 362. Association of Eosinophilia with Parasites in Rhode Island Refugees, 2015 - 2020

**DOI:** 10.1093/ofid/ofac492.440

**Published:** 2022-12-15

**Authors:** Maranatha Teferi, Marcela Osorio, Benjamin Gallo Marin, Ann Ding, Ian C Michelow

**Affiliations:** Warren Alpert Medical School of Brown University, Providence, Rhode Island; Warren Alpert Medical School at Brown University, Providence, Rhode Island; Warren Alpert Medical School of Brown University, Providence, Rhode Island; Brown University, Central Falls, Rhode Island; Connecticut Children's Medical Center, Hartford, Connecticut

## Abstract

**Background:**

There are sparse data reporting the rates of potentially pathogenic parasites in asymptomatic, newly arrived refugees to the United States. Untreated parasitic infections can have significant health consequences including anemia, malnutrition, infertility, urinary tract malignancy, and death, among others. Eosinophilia may serve as a biomarker for certain parasites, but its reliability is debated. We hypothesized that detection of eosinophilia in refugees to Rhode Island would be useful for guiding management in this vulnerable population.

**Methods:**

A retrospective chart review was performed on all adult and pediatric refugees who had their initial refugee intake clinic visit at Lifespan’s Center for Primary Care Refugee Clinic, Hasbro Children Hospital’s Refugee Clinic, or Medicine-Pediatrics Refugee Clinic, all in Rhode Island, from January 2015 to December 2020. Patients who had delayed intakes or were originally evaluated in other states were not eligible. Data were systematically collated in RedCap and descriptive statistics were performed.

**Results:**

Charts of 955 refugees were reviewed retrospectively, of which 143 did not meet eligibility criteria and were excluded. Overall, 505 (62.2%) patients were from Africa, 242 (29.8%) from Asia, 32 (3.9%) from the Americas, 32 (3.9%) from Europe, and 1 (0.1%) from Australia. Among the 812 individuals included, 147 (18.1%) patients had eosinophil counts > 500/uL, of whom 113 (76.9%) had mild (450-1499/uL), 30 (20.4%) had moderate (1500-4999/uL), and 4 (2.7%) had severe eosinophilia (⪰ 5000/uL). The majority of patients with or without eosinophilia originated from Africa.

Prevalence of symptoms (**Table 1**) ranged from 0% (bloody stools) to 17.6% (abdominal pain). Overall, > 50% of refugees tested positive for a parasite by various methods (**Table 2**). Serology did not distinguish between acute or past infection. One patient (0.7%) was diagnosed with *Plasmodium falciparum* malaria.

**Table 1.**
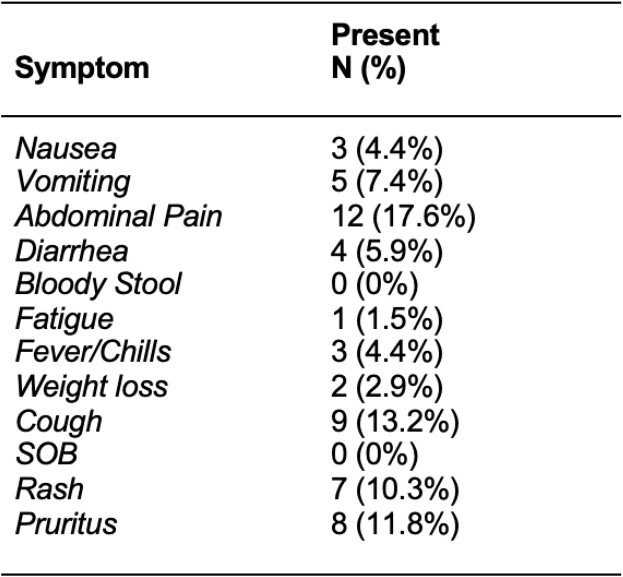
Symptoms at Initial Encounter Among Patients With Eosinophilia (n=68)

**Table 2.**
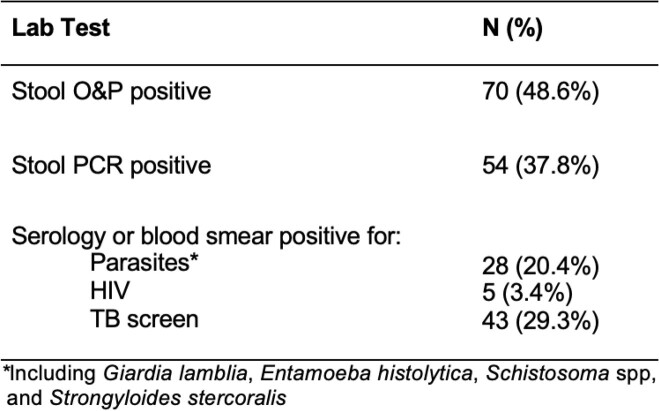
Lab Values Among Patients with Eosinophilia (n=147)

**Conclusion:**

Eosinophilia was common in both adult and pediatric asymptomatic refugees in Rhode Island who had parasites detected by various tests. Therefore, we conclude that routine testing for eosinophilia may inform treatment of potentially dangerous parasites in the absence of symptoms.

**Disclosures:**

**All Authors**: No reported disclosures.

